# Gradual extinction prevents the return of fear: implications for the discovery of state

**DOI:** 10.3389/fnbeh.2013.00164

**Published:** 2013-11-18

**Authors:** Samuel J. Gershman, Carolyn E. Jones, Kenneth A. Norman, Marie-H. Monfils, Yael Niv

**Affiliations:** ^1^Department of Brain and Cognitive Sciences, Massachusetts Institute of Technology Cambridge, MA, USA; ^2^Department of Psychology, The University of Texas at Austin Austin, TX, USA; ^3^Department of Psychology and Princeton Neuroscience Institute, Princeton University Princeton, NJ, USA

**Keywords:** extinction, Pavlovian fear conditioning, spontaneous recovery, reinstatement, memory

## Abstract

Fear memories are notoriously difficult to erase, often recovering over time. The longstanding explanation for this finding is that, in extinction training, a new memory is formed that competes with the old one for expression but does not otherwise modify it. This explanation is at odds with traditional models of learning such as Rescorla-Wagner and reinforcement learning. A possible reconciliation that was recently suggested is that extinction training leads to the inference of a new state that is different from the state that was in effect in the original training. This solution, however, raises a new question: under what conditions are new states, or new memories formed? Theoretical accounts implicate persistent large prediction errors in this process. As a test of this idea, we reasoned that careful design of the reinforcement schedule during extinction training could reduce these prediction errors enough to prevent the formation of a new memory, while still decreasing reinforcement sufficiently to drive modification of the old fear memory. In two Pavlovian fear-conditioning experiments, we show that gradually reducing the frequency of aversive stimuli, rather than eliminating them abruptly, prevents the recovery of fear. This finding has important implications for theories of state discovery in reinforcement learning.

## Introduction

Once a fear memory trace is laid down in the brain, can it be modified? When animals are conditioned to associate a cue with an aversive stimulus, repeatedly presenting the cue alone (extinction training) reduces their fear of the cue. However, this reduction is temporary, and fear generally returns with the passage of time, a phenomenon known as *spontaneous recovery* (Pavlov, 1927; Rescorla, 2004). Fear also returns following an isolated occurrence of the aversive stimulus, a phenomenon known as *reinstatement* (Pavlov, 1927; Rescorla and Heth, 1975). Rather than modifying the fear memory, it is believed that extinction training creates a new memory that only transiently inhibits the original association (Bouton, 2004).

Traditional models of Pavlovian conditioning (e.g., Rescorla and Wagner, 1972; Pearce and Hall, 1980), as well as their more modern counterparts in reinforcement learning (RL; Sutton and Barto, 1998) are at odds with the recovery of fear after extinction. These models conceive of learning as the modification of an association between each cue and the aversive stimulus; this association is strengthened during fear conditioning and then weakened during extinction. Thus, conditioned fear in a later recovery test is incorrectly predicted to be no greater than conditioned fear at the end of extinction.

One theoretical approach to this problem is to assume that animals learn a model of the environment that is richer than simple associations between cues and aversive stimuli. Bouton (2004) has suggested that animals encode the spatiotemporal context of learning, and use this to determine when and how to generalize previously learned associations to new contexts. This notion of context corresponds closely to the notion of *state* in RL (Sutton and Barto, 1998): Although no two experiences are ever identical, they can be grouped together into states that capture their statistical regularities.

One hypothesis about the persistence of fear following extinction is that the animal creates a new memory for extinction because it has discovered a new state of the world (Redish et al., 2007; Gershman et al., 2010; Gershman and Niv, 2012). In other words, the animal has (correctly) inferred that there is a “conditioning” state and an “extinction” state, and that these should be learned separately (i.e., encoded in different memories). If this is true, how might the process of state discovery be carried out in practice?

The onset of extinction training produces large “prediction errors”—discrepancies between predicted outcomes (e.g., shocks) and experienced outcomes (no shock). In RL, such prediction errors serve as a learning signal, driving the modification of predictions (Rescorla and Wagner, 1972; McNally et al., 2011). According to these accounts, the absence of shocks during extinction should reduce the strength of the original fear memory. However, recent models propose that these persistently large prediction errors might also serve as a segmentation signal, indicating to the animal a novel state that demands new associations (Redish et al., 2007; Gershman et al., 2010). This can explain why the traditional extinction procedure leads to formation of a new, competing, “no-fear” memory, all the while allowing the original fear memory to persist unmodified.

**Figure 1:**
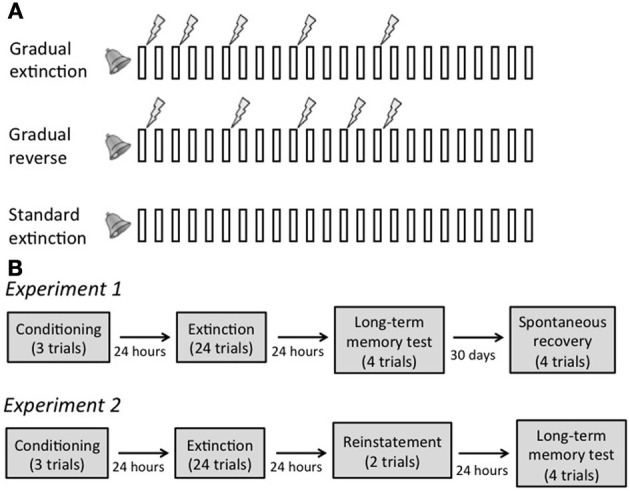
**(A)** Schematic of the extinction phase in each extinction condition. Bars represent 20 s tone presentations; lightning bolts represent 500 ms 0.7 mA foot shocks. Note that temporal relations between the stimuli are depicted for illustration only, and are not to scale. **(B)** Design of Experiments 1 and 2.

The idea that large prediction errors are a signal for state segmentation suggests that one could modify the original fear memory if prediction errors were small or infrequent enough to *not* induce formation of a new memory, but still large enough to drive learning. To test this prediction, we designed a “gradual extinction” paradigm in which the aversive event (a foot-shock) was gradually eliminated (Figure 1). The idea was to change the association of the cue from shock to no shock gradually enough so as to avoid the creation of a new memory. As a result, all learning would affect the old fear memory, which would gradually be weakened. In two experiments, we tested the hypothesis that gradual extinction would prevent the return of fear, as measured by spontaneous recovery (Experiment 1) or reinstatement (Experiment 2).

## Materials and methods

### Subjects

Subjects in both experiments were seventy-five male Sprague-Dawley rats (250–300 g; Harlan Lab Animals Inc.). Procedures were conducted in compliance with the National Institutes of Health Guide for the Care and Use of Experimental Animals and were approved by the University of Texas at Austin Animal Care and Use Committee. Rats were housed in pairs in clear plastic cages and maintained on a 12-h light/dark cycle with food and water provided *ad libitum*.

### Apparatus

Behavioral procedures took place in standard conditioning chambers equipped with metal walls and stainless-steel rod floors connected to a shock generator and enclosed in acoustic isolation boxes (Coulbourn Instruments, Allentown, PA). For each rat, all stages of the experiment took place in the same box (same context). A 20 s tone (5 kHz, 80 dB) played through a speaker in the walls of the box served as the cue (conditional stimulus), and a 500 ms 0.7 mA foot-shock served as the outcome (unconditional stimulus). Behavior was recorded using infrared digital cameras mounted on the top of each unit. Stimulus delivery was controlled using Freeze Frame software (Coulbourn Instruments).

### Procedure

The fear conditioning and extinction phases were identical in the two experiments. In the fear conditioning phase, rats were allowed to habituate to the chambers for 10 min before receiving three 20 s presentations of the tone [inter-trial intervals (ITI) = 160 and 200 s], each co-terminating with a foot-shock. All rats were then returned to their home cage.

Twenty-four hours later, the rats were divided into three extinction groups (Standard, Gradual, and Gradual Reverse) and put in the experimental chambers. Rats in the Standard group (*n* = 16 in Experiment 1 and *n* = 8 in Experiment 2) received 24 presentations of the tone in the absence of the foot-shock. Rats in the Gradual group (*n* = 16 and 12 in the two experiments, respectively) also received 24 tone presentations. However, within these, trials 1, 3, 6, 10, and 15 were paired with a foot-shock, resulting in a gradual *decrease* in the frequency of the shock. Rats in the Gradual Reverse group (*n* = 15 and 12 in the two experiments, respectively) received 24 tone presentations with trials 1, 6, 10, 13, and 15 paired with a foot-shock, resulting in a gradual *increase* in the frequency of the shock. To ensure that all groups extinguished to the same level, the last 9 tones were always presented without shock (Figure 1A). All ITIs were 160 s. After extinction, rats were returned to their home cage.

In Experiment 1 (Spontaneous Recovery), 24 h after extinction, rats were tested for long-term memory of the extinction phase by recording freezing during four presentations of the tone in the same context as conditioning and extinction. Thirty days after extinction, rats were returned to the chambers for a test of spontaneous recovery of fear by recording freezing during four presentations of the tone. Spontaneous recovery was calculated as the difference between freezing on the 4 trials of the spontaneous recovery test and the last 4 trials of extinction.

In Experiment 2 (Reinstatement), 24 h after extinction, rats were exposed to 2 unsignaled shocks, and then tested 24 h later for reinstatement of fear by recording freezing during four presentations of the tone in the same context as conditioning and extinction. Reinstatement was calculated as the difference between freezing on the 4 trials of the reinstatement test and the last 4 trials of extinction.

Freezing behavior was defined as the absence of any movement, excluding breathing and whisker twitching, and was rated manually by an observer who was unaware of the group allocation of each rat. The total number of seconds spent freezing throughout each tone presentation was then expressed as a percentage of tone duration (20 s).

## Results

### Experiment 1

**Figure 2:**
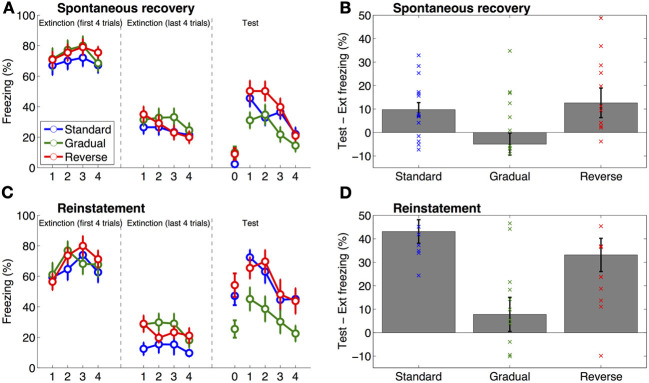
**Results of Experiments 1 and 2.** The left panels **(A,C)** show freezing on the first and last 4 trials of extinction and at test (the same data are summarized in Table 1), the right panels **(B,D)** show the difference score (% freezing) between the test phase and the end of extinction (i.e., freezing during the 4 trials of the test phase minus freezing during the last 4 trials of extinction), with individual data superimposed on the group means. Time point “0” indicates pre-tone freezing. Error bars represent standard error of the mean. **(A,B)** Results of Experiment 1, in which animals were tested for spontaneous recovery of fear 1 month after extinction. Freezing at test was greater than freezing on the last four trials of extinction (Ext) in the Standard and Gradual Reverse groups as compared to the Gradual group. **(C,D)** Results of Experiment 2, in which animals were exposed to 2 unsignaled shocks 24 h after extinction, followed by a reinstatement test 24 h later. On the reinstatement test the Standard and Gradual Reverse groups froze significantly more than the Gradual group.

There was no significant difference between the three groups in terms of levels of freezing on the first four trials of extinction (One-Way ANOVA, *P* = 0.37, Figure 2A) or on the last four trials of extinction (One-Way ANOVA, *P* = 0.50, Figure 2A). However, there was a significant effect of group on freezing on the long-term memory test on the next day [One-Way ANOVA, *F*_(2, 44)_ = 11.67, *P* < 0.001]. This effect was driven by significantly lower freezing in the Standard group compared to the Gradual and Gradual Reverse groups [*F*_(1, 44)_ = 19.42 for the contrast of Standard vs. both other groups, *P* < 0.001], which may reflect a lesser degree of extinction in the latter groups. There was a similar significant effect of group on the difference between freezing on the long-term memory test and freezing at the end of extinction [One-Way ANOVA, *F*_(2, 44)_ = 9.31, *P* < 0.001]. We note that the difference between groups on the long-term memory test does not pose a confound for our hypothesis as it makes it more likely that fear would recover at test compared to the end of extinction in both these groups.

Pre-tone freezing in the spontaneous recovery test was not significantly different between the groups (One-Way ANOVA, *P* = 0.13; Figure 2A). Thus, there was no evidence for differences in general fear of the context (the box). However, as predicted, there was a significant effect of group on freezing to the tone in the spontaneous recovery test [One-Way ANOVA, *F*_(2, 44)_ = 3.26, *P* < 0.05; Figure 2A and **Table 2**]. A planned contrast showed that rats in the Gradual group froze to the tone significantly less than rats in the Standard and Gradual Reverse group [*F*_(1, 44)_ = 5.51, *P* < 0.05]. Similarly, there was a significant effect of group on the difference between freezing on the spontaneous recovery test and the last 4 trials of extinction [One-Way ANOVA, *F*_(2, 44)_ = 3.91, *P* < 0.05; Figure 2B]. A planned contrast (2 × Gradual - Standard - Gradual Reverse) showed that the difference score for the Gradual group was significantly lower than for the Standard and Gradual Reverse groups [*F*_(1, 44)_ = 7.67, *P* < 0.01]. Each of these comparisons was also significant separately: The difference score for the Gradual group was significantly lower than for the Standard group [*t*_(30)_ = 2.64, *P* < 0.05] as well as for the Gradual Reverse group [*t*_(29)_ = 2.26, *P* < 0.05].

### Experiment 2

Similarly to Experiment 1, in Experiment 2 we again observed no significant differences between groups in terms of the levels of freezing on the first four trials of extinction (One-Way ANOVA, *P* = 0.79, Figure 2C) or on the last four trials of extinction (One-Way ANOVA, *P* = 0.07, Figure 2C), although numerically there was less freezing in the Standard group (Table 1).

**Table 1:** **Average freezing and 95% confidence intervals on the last 4 trials of extinction in Experiments 1 and 2**.

	**Experiment 1 (Spontaneous recovery)**		**Experiment 2 (Reinstatement)**	
	**Freezing (%)**	**95% confidence interval (%)**	**Freezing (%)**	**95% confidence interval (%)**
Standard	24	18–31	13	4–22
Gradual	30	22–39	26	17–36
Reverse	27	20–34	23	17–30

Of main interest was the reinstatement test, 1 day after the two unpaired reminder shocks. Pre-tone freezing in the reinstatement test was significantly different between the groups [One-Way ANOVA, *F*_(2, 29)_ = 5.41, *P* < 0.05; Figure 2C]. A planned contrast showed that rats in the Gradual group froze significantly less than in the Standard and Gradual Reverse group [*F*_(1, 29)_ = 9.63, *P* < 0.01], suggesting that the Standard and Gradual Reverse groups preserved some contextual fear following extinction. Moreover, as predicted, there was a significant effect of group on freezing to the tone in the reinstatement test [One-Way ANOVA, *F*_(2, 29)_ = 4.04, *P* < 0.05; Figure 2C and Table 2]. A planned contrast showed that rats in the Gradual group froze to the tone significantly less than in the Standard and Gradual Reverse group [*F*_(1, 29)_ = 7.94, *P* < 0.01]. This difference was also manifest in the difference scores: there was a significant effect of group on freezing on the difference between freezing on the 4 trials of the reinstatement test and the last 4 trials of extinction [One-Way ANOVA, *F*_(2, 29)_ = 6.70, *P* < 0.005; Figure 2D]. A planned comparison showed that the difference score for the Gradual group was significantly lower than that for the Standard and Gradual Reverse groups [*F*_(1, 29)_ = 13.13, *P* < 0.005]. In summary, these results demonstrate heightened fear (both to the context and to the tone) in the Standard and Gradual Reverse groups, as compared to the Gradual group, in accordance with our predictions.

**Table 2:** **Average freezing and 95% confidence intervals on the test trials in Experiments 1 and 2**.

	**Experiment 1 (Spontaneous recovery)**		**Experiment 2 (Reinstatement)**	
	**Freezing (%)**	**95% confidence interval (%)**	**Freezing (%)**	**95% confidence interval (%)**
Standard	28	21–35	54	45–64
Gradual	22	16–29	32	20–45
Reverse	34	25–43	56	40–72

*In Experiment 1, the test trials occurred one month after extinction. In Experiment 2, the test trials occurred 48 h after extinction.*

## Discussion

We found that gradually reducing the tone-shock contingency during extinction was effective in preventing the subsequent return of fear. This contrasted with regular extinction and a “gradual reverse” control (in which the tone-shock contingency was gradually increased during extinction): Both of these extinction protocols were ineffective at persistently decreasing the conditioned response, as measured by spontaneous recovery (Experiment 1) and reinstatement (Experiment 2).

Our results fit well with emerging theoretical ideas about the role of state discovery in Pavlovian conditioning and other learning paradigms (Redish et al., 2007; Gershman et al., 2010; Gershman and Niv, 2012). In traditional models (e.g., Rescorla and Wagner, 1972), one association is learned for each cue-outcome pair. As mentioned, such models typically have trouble dealing with fear recovery phenomena (though see Larrauri and Schmajuk, 2006), because in extinction the models unlearn the association acquired during conditioning. In contrast, we have proposed in our previous work (Gershman et al., 2010; Gershman and Niv, 2012) that animals infer the possibly unobservable states that give rise to sensory data—formalized probabilistically as “latent causes” (see also Courville et al., 2006).

According to our theory, when conditions change considerably, such as when transitioning from reinforced trials in acquisition to non-reinforced trials in extinction, the animal infers that a new latent cause is responsible for the new observed data^1^. From a statistical modeling perspective, the animal's inference here is correct—the causal structure of the environment actually is, in fact, different in the acquisition phase vs. the extinction phase—in the former, the experimenter causes tones to be followed by shocks, whereas in the latter the experimenter causes tones to appear without shocks. Inferring a new latent cause is equivalent to learning a new association, segmenting a new state of the task, or storing a new memory—concepts that are united in our theory. Moreover, our theory is similar to accounts that rely on the notion of “context” as a construct that groups associations together, although our theory generalizes the concept of context to situations that are perceptually similar (the animal is in the same experimental box) but nevertheless correspond to different causal structures of the environment (see also Redish et al., 2007).

Importantly, in our theory, the animal's belief about whether a particular latent cause is currently active is determined by the similarity between the current situation and those that occurred when the latent cause was previously active. This explains why abrupt extinction, in which conditions change dramatically, brings about inference of a new latent cause and learning of a new associative weight. By titrating the similarity between the extinction and acquisition situations, and only gradually moving away from the acquisition situation, we endeavored to prevent the inference of a new latent cause; this way, new experience would result in modification (gradual weakening) of the association learned during the acquisition phase.

Our results are consistent with several previous studies that examined the effect of partial reinforcement during extinction. Bouton et al. (2004) also found that a gradual extinction procedure (in which reinforcement was gradually reduced across sessions) was effective at slowing reacquisition, thus confirming the predictions of our model. Bouton et al.'s experiments differed from our own in several ways: They used appetitive instead of aversive conditioning, their gradual reductions were performed across sessions rather than within a session, and they used the speed of reacquisition to measure preservation of the original memory. Because a reacquisition test involves new learning, it is difficult to isolate how their experimental procedure affects the original fear memory as opposed to subsequent learning. Nevertheless, their results are fully consistent with our theoretical account of gradual extinction and suggest that our theory should generalize to the appetitive domain as well.

Using the rabbit nictitating membrane preparation, Kehoe and White (2002) showed that gradual reductions in unconditioned stimulus intensity produced proportional reductions in the conditioned response. However, they found between-session spontaneous recovery, indicating that their procedure did not persistently attenuate the conditioned response. One potential reason for the discrepancy with our results is that Kehoe and White reduced the intensity, rather than frequency, of the unconditioned stimulus. If the subjective perception of aversive stimuli is not a linear function of their intensity, gradual reductions in intensity may still result in the experience of an abrupt change, generating a segmentation signal that leads to formation of a new memory trace. This hypothesis needs to be tested in future studies.

Our findings are also consistent with the effects of partial reinforcement during conditioning. One of the most well-known findings in Pavlovian conditioning is the *partial reinforcement extinction effect* (PREE): extinction is slower if the cue was reinforced only on some of the trials during conditioning, compared to the standard condition in which the cue is always reinforced. As pointed out by others (Capaldi, 1957; Gallistel and Gibbon, 2000; Courville et al., 2006), this finding can be rationalized by assuming that partial reinforcement renders extinction less discriminable from conditioning. In the language of RL, partial reinforcement obscures the differences between the conditioning and extinction states. Redish et al. (2007) showed that an RL agent that uses reinforcement history (time since last reinforcement) to discriminate between states will indeed exhibit the PREE. If the PREE is due to inference of a single latent cause for both acquisition and extinction, we would predict that partial reinforcement during conditioning should also attenuate spontaneous recovery and reinstatement. A similar effect can be achieved by making extinction less discriminable from a subsequent test or reacquisition phase. For example, Winstanley et al. (2011) found that “near-misses” (i.e., unrewarded trials on which the rat has a high expectation of reward) during extinction result in slower reacquisition compared to a group in which near-misses were absent during extinction. Because near-misses occurred in the reacquisition phase, the two phases were less discriminable when extinction included near-misses.

However, we note that the gradual extinction effect is distinct from the PREE. In our protocol, during extinction the probability of reinforcement changed dynamically over the course of the session. Comparison between the Gradual and Gradual Reverse conditions shows that the overall rate of reinforcement in extinction was not the determinant of the rate of reinstatement or spontaneous recovery, as both groups had the same overall rate of reinforcement in the extinction phase. What distinguishes the Gradual and Gradual Reverse conditions is the direction in which the reinforcement probability changed: in the former reinforcement became less and less frequent, and vice versa in the latter. As such, we believe that a successful computational account of our data must be augmented with knowledge of how reinforcement probabilities change over time (see Courville et al., 2006).

In summary, our experimental results demonstrate the paradoxical effect that *more* tone-shock pairs (in extinction) can result in *reduced* return of fear. Importantly, our results cannot be attributed simply to partial reinforcement during extinction: a Gradual Reverse control condition, in which the tone and shock were paired the same number of times as in the Gradual condition but with increasing frequency, led to the return of fear. We interpret our results as showing that gradually reducing the frequency of tone-shock pairs prevents the formation of a new memory and thus leads to gradual modification of the original memory. Gradual extinction can therefore be added to the toolbox of behavioral (e.g., Monfils et al., 2009) and neural (e.g., Nader et al., 2000) techniques for modifying memories. More broadly, our experimental results provide hints about how the brain discovers new states. Linking memory formation to state discovery in RL may provide a new path toward a quantitative theory of memory modification.

### Conflict of interest statement

The authors declare that the research was conducted in the absence of any commercial or financial relationships that could be construed as a potential conflict of interest.

## Footnotes

^1^Note that in our probabilistic theory, there is not a threshold on the magnitude of prediction errors that can cause state segmentation. Given a large prediction error, the animal would be more likely to infer a new latent cause than if only a small error occurred. In both cases, however, prediction errors are used for learning in addition to their possible contribution to state segmentation.

## References

[B1] Bouton M. E. (2004). Context and behavioral processes in extinction. Learn. Mem. 11, 485–494. 10.1101/lm.78804 15466298

[B2] Bouton M. E. Woods A. M. Pineño O. (2004). Occasional reinforced trials during extinction can slow the rate of rapid reacquisition. Learn. Motiv. 35, 371–390. 10.1016/j.lmot.2004.05.001 19132143 PMC2614821

[B3] Capaldi E. J. (1957). The effect of different amounts of alternating reinforcement on resistance to extinction. Am. J. Psychol. 70, 451–452. 10.2307/1419584 13458520

[B4] Courville A. C. Daw N. D. Touretzky D. S. (2006). Bayesian theories of conditioning in a changing world. Trends Cogn. Sci. 10, 294–300. 10.1016/j.tics.2006.05.004 16793323

[B5] Gallistel C. R. Gibbon J. (2000). Time, rate, and conditioning. Psychol. Rev. 107, 289–344. 10.1037/0033-295X.107.2.289 10789198

[B6] Gershman S. J. Blei D. M. Niv Y. (2010). Context, learning, and extinction. Psychol. Rev 117, 197–209. 10.1037/a0017808 20063968

[B8] Gershman S. J. Niv Y. (2012). Exploring a latent cause model of classical conditioning. Learn. Behav. 40, 255–268. 10.3758/s13420-012-0080-8 22927000

[B9] Kehoe E. J. White N. E. (2002). Extinction revisited: similarities between extinction and reductions in US intensity in classical conditioning of the rabbit's nictitating membrane response. Anim. Learn. Behav. 30, 96–111. 10.3758/BF03192912 12141139

[B10] Larrauri J. A. Schmajuk N. A. (2006). Attentional, associative, and configural mechanisms in extinction. Psych. Rev. 115, 640–676. 10.1037/0033-295X.115.3.640 18729595

[B11] McNally G. P. Johansen J. P. Blair H. T. (2011). Placing prediction into the fear circuit. Trends Neurosci. 34, 283–292. 10.1016/j.tins.2011.03.005 21549434 PMC4245078

[B12] Monfils M. H. Cowansage K. K. Klann E. LeDoux J. E. (2009). Extinction-reconsolidation boundaries: key to persistent attenuation of fear memories. Science 324, 951–955. 10.1126/science.1167975 19342552 PMC3625935

[B13] Nader K. Schafe G. E. LeDoux J. E. (2000). Fear memories require protein synthesis in the amygdala for reconsolidation after retrieval. Nature 406, 722–726. 10.1038/35021052 10963596

[B15] Pavlov I. V. (1927). Conditioned Reflexes, Trans. Anrep G. V. (New York, NY: Liveright).

[B16] Pearce J. M. Hall G. (1980). A model for Pavlovian learning: variations in the effectiveness of conditioned but not of unconditioned stimuli. Psychol. Rev. 87, 532–552. 10.1037/0033-295X.87.6.532 7443916

[B17] Redish A. D. Jensen S. Johnson A. Kurth-Nelson Z. (2007). Reconciling reinforcement learning models with behavioral extinction and renewal: implications for addiction, relapse, and problem gambling. Psych. Rev. 114, 784–805. 10.1037/0033-295X.114.3.784 17638506

[B18] Rescorla R. A. (2004). Spontaneous recovery. Learn. Mem. 11, 501–509. 10.1101/lm.77504 15466300

[B19] Rescorla R. A. Heth C. D. (1975). Reinstatement of fear to an extinguished conditioned stimulus. J. Exp. Psychol. Anim. Behav. Process. 1, 88–96. 10.1037/0097-7403.1.1.88 1151290

[B20] Rescorla R. A. Wagner A. R. (1972). A theory of Pavlovian conditioning: variations in the effectiveness of reinforcement and nonreinforcement, in Classical Conditioning II: Current Research and Theory, eds Black A. H. Prokasy W. F. (New York, NY: Appleton-Century Crofts), 64–69.

[B21] Sutton R. Barto A. (1998). Reinforcement Learning: An Introduction. Cambridge, MA: MIT Press.

[B21a] Winstanley C. A. Cocker P. J. Rogers R. D. (2011). Dopamine modulates reward expectancy during performance of a slot machine task in rats: evidence for a ‘near-miss’ effect. Neuropsychopharmacology 36, 913–925. 10.1038/npp.2010.230 21209612 PMC3077261

