# Corrigendum: Gradual extinction prevents the return of fear: implications for the discovery of state

**DOI:** 10.3389/fnbeh.2021.786900

**Published:** 2021-11-29

**Authors:** Samuel J. Gershman, Carolyn E. Jones, Kenneth A. Norman, Marie-H. Monfils, Yael Niv

**Affiliations:** ^1^Department of Brain and Cognitive Sciences, Massachusetts Institute of Technology, Cambridge, MA, USA; ^2^Department of Psychology, The University of Texas at Austin, Austin, TX, USA; ^3^Department of Psychology and Princeton Neuroscience Institute, Princeton University, Princeton, NJ, USA

**Keywords:** extinction, Pavlovian fear conditioning, spontaneous recovery, reinstatement, memory

There was an analysis error in the original article. One animal in Experiment 1 that did not have data for the spontaneous recovery test was erroneously included in the data analysis. We have removed this animal and reported correct statistics below. In addition, the degrees of freedom for some ANOVAs were reported incorrectly.

There is one change in the new statistical results: we had originally reported that the difference score comparing freezing in the long-term memory test and the end of extinction in Experiment 1 did not vary significantly across groups; however, there was in fact a significant effect, driven by an increase in freezing rate for both gradual extinction and gradual reverse groups in the long-term memory test (compared to the end of extinction), but not for the standard extinction group. Our key result, that there was no significant spontaneous recovery for the gradual extinction group when comparing freezing at test to the end of extinction (not to the long-term memory test), remains unchanged.

A correction has been made to ***Results***, **Experiment 1**. The corrected Experiment 1 section is below:

There was no significant difference between the three groups in terms of levels of freezing on the first four trials of extinction (One-Way ANOVA, *P* = 0.37, **Figure 2A**) or on the last four trials of extinction (One-Way ANOVA, *P* = 0.50, **Figure 2A**). However, there was a significant effect of group on freezing on the long-term memory test on the next day [One-Way ANOVA, *F*_(2, 44)_ = 11.67, *P* < 0.001]. This effect was driven by significantly lower freezing in the Standard group compared to the Gradual and Gradual Reverse groups [*F*_(1, 44)_ = 19.42 for the contrast of Standard vs. both other groups, *P* < 0.001], which may reflect a lesser degree of extinction in the latter groups. There was a similar significant effect of group on the difference between freezing on the long-term memory test and freezing at the end of extinction [One-Way ANOVA, *F*_(2, 44)_ = 9.31, *P* < 0.001]. We note that the difference between groups on the long-term memory test does not pose a confound for our hypothesis as it makes it more likely that fear would recover at test compared to the end of extinction in both these groups.

Pre-tone freezing in the spontaneous recovery test was not significantly different between the groups (One-Way ANOVA, *P* = 0.13; **Figure 2A**). Thus, there was no evidence for differences in general fear of the context (the box). However, as predicted, there was a significant effect of group on freezing to the tone in the spontaneous recovery test [One-Way ANOVA, *F*_(2, 44)_ = 3.26, *P* < 0.05; **Figure 2A** and **Table 2**]. A planned contrast showed that rats in the Gradual group froze to the tone significantly less than rats in the Standard and Gradual Reverse group [*F*_(1, 44)_ = 5.51, *P* < 0.05]. Similarly, there was a significant effect of group on the difference between freezing on the spontaneous recovery test and the last 4 trials of extinction [One-Way ANOVA, *F*_(2, 44)_ = 3.91, *P* < 0.05; **Figure 2B**]. A planned contrast (2 × Gradual - Standard - Gradual Reverse) showed that the difference score for the Gradual group was significantly lower than for the Standard and Gradual Reverse groups [*F*_(1, 44)_ = 7.67, *P* < 0.01]. Each of these comparisons was also significant separately: The difference score for the Gradual group was significantly lower than for the Standard group [*t*_(30)_ = 2.64, *P* < 0.05] as well as for the Gradual Reverse group [*t*_(29)_ = 2.26, *P* < 0.05].

A correction has been made to ***Results***, **Experiment 2**. The corrected Experiment 2 section is below:

Similarly to Experiment 1, in Experiment 2 we again observed no significant differences between groups in terms of the levels of freezing on the first four trials of extinction (One-Way ANOVA, *P* = 0.79, **Figure 2C**) or on the last four trials of extinction (One-Way ANOVA, *P* = 0.07, **Figure 2C**), although numerically there was less freezing in the Standard group (**Table 1**).

Of main interest was the reinstatement test, 1 day after the two unpaired reminder shocks. Pre-tone freezing in the reinstatement test was significantly different between the groups [One-Way ANOVA, *F*_(2, 29)_ = 5.41, *P* < 0.05; **Figure 2C**]. A planned contrast showed that rats in the Gradual group froze significantly less than in the Standard and Gradual Reverse group [*F*_(1, 29)_ = 9.63, *P* < 0.01], suggesting that the Standard and Gradual Reverse groups preserved some contextual fear following extinction. Moreover, as predicted, there was a significant effect of group on freezing to the tone in the reinstatement test [One-Way ANOVA, *F*_(2, 29)_ = 4.04, *P* < 0.05; **Figure 2C** and **Table 2**]. A planned contrast showed that rats in the Gradual group froze to the tone significantly less than in the Standard and Gradual Reverse group [*F*_(1, 29)_ = 7.94, *P* < 0.01]. This difference was also manifest in the difference scores: there was a significant effect of group on freezing on the difference between freezing on the 4 trials of the reinstatement test and the last 4 trials of extinction [One-Way ANOVA, *F*_(2, 29)_ = 6.70, *P* < 0.005; **Figure 2D**]. A planned comparison showed that the difference score for the Gradual group was significantly lower than that for the Standard and Gradual Reverse groups [*F*_(1, 29)_ = 13.13, *P* < 0.005]. In summary, these results demonstrate heightened fear (both to the context and to the tone) in the Standard and Gradual Reverse groups, as compared to the Gradual group, in accordance with our predictions.

The authors apologize for this error. The original article has been updated.

## Publisher's Note

All claims expressed in this article are solely those of the authors and do not necessarily represent those of their affiliated organizations, or those of the publisher, the editors and the reviewers. Any product that may be evaluated in this article, or claim that may be made by its manufacturer, is not guaranteed or endorsed by the publisher.

